# Detecting Alzheimer’s disease biomarkers with a brief tablet-based cognitive battery: sensitivity to Aβ and tau PET

**DOI:** 10.1186/s13195-021-00776-w

**Published:** 2021-02-08

**Authors:** Elena Tsoy, Amelia Strom, Leonardo Iaccarino, Sabrina J. Erlhoff, Collette A. Goode, Anne-Marie Rodriguez, Gil D. Rabinovici, Bruce L. Miller, Joel H. Kramer, Katherine P. Rankin, Renaud La Joie, Katherine L. Possin

**Affiliations:** 1 Department of Neurology, Memory and Aging Center, University of California San Francisco, Box 1207, 675 Nelson Rising Lane, Suite 190, San Francisco, CA 94158 USA; 2 Department of Radiology and Biomedical Imaging, University of California San Francisco, 1500 Owens Street, 2nd Fl, San Francisco, CA 94158 USA; 3 Global Brain Health Institute, University of California San Francisco, 675 Nelson Rising Lane, San Francisco, CA 94158 USA

**Keywords:** Alzheimer’s disease, Mild cognitive impairment, Neuropsychology, Psychometrics, Positron emission tomography, Biomarkers

## Abstract

**Background:**

β-amyloid (Aβ) and tau positron emission tomography (PET) detect the pathological changes that define Alzheimer’s disease (AD) in living people. Cognitive measures sensitive to Aβ and tau burden may help streamline identification of cases for confirmatory AD biomarker testing.

**Methods:**

We examined the association of Brain Health Assessment (BHA) tablet-based cognitive measures with dichotomized Aβ -PET status using logistic regression models in individuals with mild cognitive impairment (MCI) or dementia (*N* = 140; 43 Aβ-, 97 Aβ+). We also investigated the relationship between the BHA tests and regional patterns of tau-PET signal using voxel-wise regression analyses in a subsample of 60 Aβ+ individuals with MCI or dementia.

**Results:**

Favorites (associative memory), Match (executive functions and speed), and Everyday Cognition Scale scores were significantly associated with Aβ positivity (area under the curve [AUC] = 0.75 [95% CI 0.66–0.85]). We found significant associations with tau-PET signal in mesial temporal regions for Favorites, frontoparietal regions for Match, and occipitoparietal regions for Line Orientation (visuospatial skills) in a subsample of individuals with MCI and dementia.

**Conclusion:**

The BHA measures are significantly associated with both Aβ and regional tau in vivo imaging markers and could be used for the identification of patients with suspected AD pathology in clinical practice.

**Supplementary Information:**

The online version contains supplementary material available at 10.1186/s13195-021-00776-w.

## Introduction

Alzheimer’s disease (AD) is a major cause of dementia in older adults. The disease is defined by abnormal accumulation of two proteins: fibrillar β-amyloid (Aβ) peptides and phosphorylated neurofibrillary tau deposits [1]. Recent developments of in vivo molecular imaging modalities have made it possible to detect underlying pathological changes associated with AD [2]. Both Aβ and tau positron emission tomography (PET) biomarkers are included as defining features of AD in the National Institute on Aging and Alzheimer’s Association (NIA-AA) research framework [3] and have been approved for clinical use by the U.S. Food and Drug Administration [4, 5]. As the prevalence of AD continues to rise, development of effective diagnostic markers and approaches is critical for diagnostic accuracy and identification of candidates for clinical trials and disease-modifying therapies on the horizon [6].

Several studies have investigated the association between cognitive measures and AD PET pathology markers [7–11]. Specifically, past studies have found moderate to strong associations between tau-PET and cognition suggesting that the topography of tau tracer binding corresponds with cognitive performance in the domains associated with both typical [11] and atypical [12–14] AD. Additionally, tau-PET has been associated with cognitive performance and decline in cognitively normal older adults [9, 15, 16] as well as with the severity of functional impairment in mixed clinical samples [17, 18]. Similarly, associations between Aβ -PET burden and cognition have been reported in both clinically mixed [10, 11, 19–23] and cognitively unimpaired [24, 25] samples, including greater rates of decline in cognitively normal Aβ-positive (Aβ+) older individuals [24, 25]. However, the effects of greater Aβ burden on cognitive performance tend to be weaker and less specific compared to tau [11, 25] likely due to the fact that tau pathology is more strongly related to neuronal loss in affected brain areas [26].

At the same time, PET studies remain largely cost-prohibitive for widescale use and the need for brief and efficient tools for the detection of AD pathology remains [27, 28]. Given the association of in vivo markers with cognition, brief, reliable, and sensitive cognitive measures have the potential to address this need as a frontline cost-effective clinical marker [27]. Time- and cost-effective clinical markers that are strongly associated with Aβ and tau markers may help significantly reduce the need for PET scans in clinic and enable multimodal case identification as a scalable alternative to lengthy clinical and diagnostic evaluations. These non-invasive frontline measures would not replace comprehensive clinical and neuropsychological assessments and standard imaging and laboratory tests but would rather enhance clinical efficiency of these diagnostic studies by offering providers a means for determining who needs referrals for comprehensive assessment for diagnostic confirmation [28]. Additionally, robust multidomain measures of cognitive functions would enhance the implementation of PET results for clinical implications and care pathways, particularly in the light of past evidence on positive PET findings in cognitively unimpaired individuals [9, 15, 16, 24, 25].

In this study, we explored the associations between cognitive performance on the University of California San Francisco (UCSF) Brain Health Assessment (BHA), a brief tablet-based battery developed and validated for the detection of neurocognitive disorders in older adults [29]. In addition to its brevity and advantages as a computerized cognitive measure, the BHA strengths include neuroanatomical validity of each of its novel constituent tasks [29], availability of regression-based norms for English and Spanish speakers [30], and a robust global cognitive composite which reliably measures cognitive change over time [30]. The BHA has also been previously shown to be sensitive to longitudinal cognitive decline in Aβ+ cognitively normal older adults compared to their Aβ− counterparts [30]. This study expands on those findings by examining the associations between BHA performance and Aβ and tau PET burden in a clinically heterogeneous sample of older adults with mild cognitive impairment (MCI) and dementia. We hypothesized that performance on the novel BHA tests would be significantly associated with Aβ+ status, and with regional tau signal in mesial temporal region for Favorites (associative memory), frontal and parietal regions for Match (executive functions and processing speed), and occipital and parietal regions for Line Orientation (visuospatial skills).

## Methods

### Participants

**Fig. 1:**
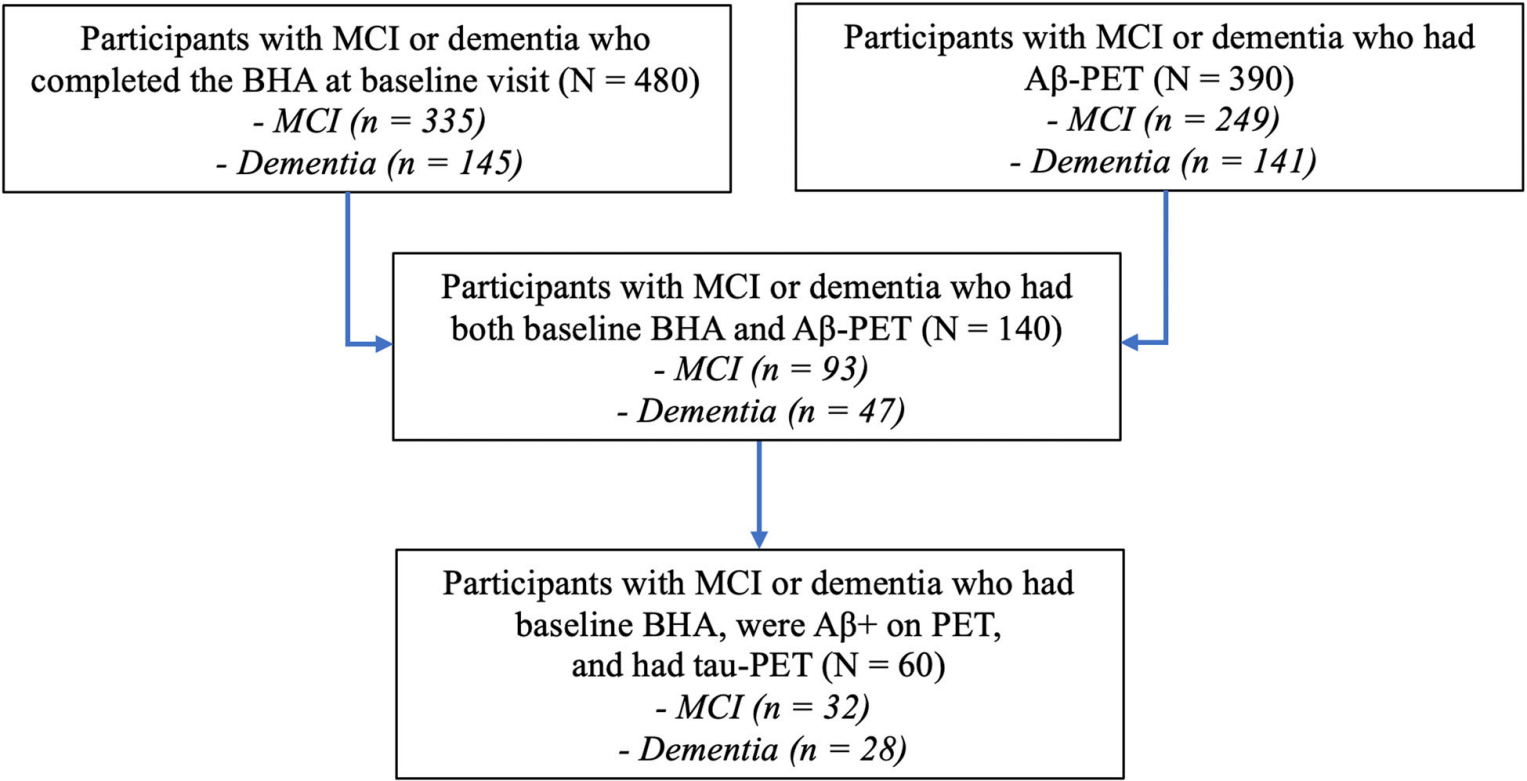
Sample selection flowchart. Abbreviations: BHA, Brain Health Assessment

The study was approved by the UCSF and the University of California Berkeley Committees on Human Research and Lawrence Berkeley National Laboratory (LBNL) Human Subject Committee, and all participants provided written informed consent. Participants were English-speaking older adults aged 50 or older and were recruited from longitudinal observational studies at the UCSF Memory and Aging Center. All participants underwent a comprehensive diagnostic evaluation including neurological and neuropsychological examination, clinical interview with an informant including Clinical Dementia Rating (CDR [31]), and structural neuroimaging. The final diagnoses were made in multidisciplinary clinical consensus conferences based on published criteria as previously described [29, 30]. Participants were included in this study if they had a diagnosis of MCI or dementia, completed the BHA tests, and underwent Aβ -PET imaging in addition to standard diagnostic evaluations (*N* = 140, Fig. 1). MCI participants were classified as amnestic based on presence at least 2 of the following characteristics: subjective report of memory problems, informant report of memory problems on CDR, or disproportionately poor performance on tests of memory on the standard neuropsychological battery described elsewhere [12], all other MCI participants were classified as non-amnestic, and dementia participants were classified as amnestic-predominant or atypical (including non-amnestic AD-type dementia or another non-AD-type dementia syndrome) based on published criteria as previously described [29, 30]. The tau-PET sample was comprised of a subgroup of participants with Aβ -PET who met all of the aforementioned criteria and were also found to be Aβ+ on PET (*N* = 60, Fig. 1). Aβ− and tau-PET results were not used to inform clinical diagnoses at any point. Exclusion criteria were presence of severe psychiatric illness, other non-neurodegenerative neurological condition that could affect cognition, substance use disorder diagnosed in the last 20 years, or significant systemic illness.

### Measures

### Cognitive tests

The BHA is a 10-min tablet-based cognitive battery programmed in the TabCAT software platform (UCSF, San Francisco, CA). The battery is comprised of 4 subtests, including 3 novel tasks which were included in this study: Favorites (associative memory), Match (executive functioning and processing speed), and Line Orientation (visuospatial skills) [29, 30]. The optional Animal Fluency subtest was not included because it has been previously examined in relation to biomarkers in similar clinical cohorts [8, 12]. The BHA also includes an optional informant-facing functional survey, the Brain Health Survey (BHS), which includes the short form of the Everyday Cognition Scales (BHS-ECog) [32]. Detailed task and survey descriptions were previously published [29, 30] and are available at memory.ucsf.edu/tabcat. Participants completed the BHA on a 9.7-in. iPad with a trained examiner in a private examination room. A subset of participants (*n* = 109, Supplementary Table 1) also completed the Montreal Cognitive Assessment (MoCA) [33] which is a widely used brief paper-and-pencil measure. Both the BHA and the MoCA were administered independent of diagnostic assessments and PET findings.

### PET acquisition

Participants included in the Aβ analyses underwent Aβ PET imaging up to 6 months pre- or up to 3 years post-BHA administration. For the tau analyses, participants underwent 18F-Flortaucipir (tau) PET imaging up to 6 months pre- or up to 1 year post-BHA administration. Aβ imaging was based on PET with either 18F-Florbetapir (*n* = 35) or 11C-Pittsburgh Compound B (PIB; *n* = 105). Florbetapir imaging was acquired on a GE Discovery STE/VCT PET-CT scanner at UCSF (*n* = 34) or a Siemens Biograph 6 Truepoint PET/CT scanner at the LBNL (*n* = 1). PIB imaging was acquired on a Siemens Biograph 6 Truepoint PET/CT scanner at the LBNL (*n* = 105). Florbetapir acquisition and processing was performed in accordance with the Alzheimer’s Disease Neuroimaging Initiative (ADNI) protocol [34]. Briefly, participants were scanned from 50 to 70 min post-injection of 10 mCi of Florbetapir. PET frames were smoothed and averaged to achieve an effective 8 × 8 × 8 mm resolution. The whole cerebellum was used as the reference region to create standard uptake value ratio (SUVR) images. PIB and Flortaucipir imaging was performed on the Siemens Biograph at LBNL in 3D acquisition mode. A low-dose CT scan was acquired for attenuation correction. Participants were scanned from 50 to 70 min post-injection of 15 mCi of PIB and from 80 to 100 min post-injection of 10 mCi of Flortaucipir. Both tracers were synthesized and radiolabeled at the LBNL Biomedical Isotope Facility. Data were reconstructed using an ordered subset expectation maximization algorithm and smoothed with a 4-mm Gaussian kernel with scatter correction. Resulting PET frames were realigned, averaged, and coregistered to the participant’s MRI to create an SUVR image using the inferior cerebellar gray as the reference region to avoid contamination from off-target binding in the dorsal cerebellum for Flortaucipir PET [35] and the cerebellar gray for PIB. Aβ status was determined by visual assessment of PIB/Florbetapir SUVR images by an expert neurologist.

To examine the associations of cognitive performance and tau retention, voxel-wise regressions were performed for each of the novel BHA tests and Flortaucipir SUVR. To prepare Flortaucipir PET for voxel-wise analyses, SUVR images were warped to Montreal Neurological Institute (MNI) space following the MRI-based deformation parameters using SPM12 (fil.ion.ucl.ac.uk/spm/software/spm12/). Warped images were then smoothed using a 4-mm FWHM Gaussian kernel within a mask that excluded extracerebral voxels using the AFNI 3dBlurInMask command.

### Statistical analyses

Differences in demographic characteristics between Aβ− and Aβ+ groups were compared based on independent sample *t*-tests for continuous variables and Fisher’s exact tests for categorical variables. Raw scores on the BHA subtests were converted to demographically adjusted (age, sex, education) *z*-scores as previously described [30]. BHS-ECog scores were included as average values derived from responses on 12 ECog questions [32].

For Aβ analyses, we performed logistic regression models to investigate the relationship between dichotomous Aβ status outcome (dummy-coded: 0 = Aβ−, 1 = Aβ+) and performance on each of the novel BHA tests. All models included covariates for age (years), sex (dummy-coded: 0 = male, 1 = female), education (years), disease severity (CDR Sum of Boxes), clinical phenotype (dummy-coded: 0 = non-amnestic MCI/atypical dementia, 1 = amnestic MCI/amnestic-predominant dementia), and time difference between PET acquisition and BHA completion. We performed receiver operating characteristic (ROC) analyses to examine the predictive accuracy of the BHA tests with (adjusted) and without (unadjusted) inclusion of demographic and clinical characteristics. The selection of variables for ROC curve analyses was informed by the results of the logistic regression and only significant predictors were chosen. We also conducted log likelihood ratio tests to compare the goodness of fit of adjusted and unadjusted models to facilitate interpretation of results. In sensitivity analyses, we repeated all primary analyses using demographically unadjusted raw scores on the BHA tests. Additionally, we performed supplementary logistic regression analyses using MoCA total score as a predictor of Aβ positivity.

For tau analyses, voxel-wise analyses were performed in SPM12 within a cortical gray matter mask, with and without inclusion of age as a covariate, as age has been previously found to be strongly associated with cortical tau burden [36]. In sensitivity analyses, we included additional covariates for sex and education. Voxel-wise analyses were thresholded using 2 approaches. First, a relatively liberal threshold consisted of an uncorrected *P* < .001 at the voxel level combined with a cluster extent of 100 voxels. Second, a more stringent family-wise error (FWE)-corrected *P* < .05 voxel level threshold was applied. Thresholded SPM T-maps were surface-rendered with BrainNet Viewer [37]. In addition, unthresholded statistical maps corresponding to all voxel-wise figure panels are freely available for viewing or download on Neurovault (https://neurovault.org/collections/FEEVNTPD/). Additionally, we fit multiple linear regression models to investigate the association between Flortaucipir SUVR in significant clusters (at *P* < .001 uncorrected threshold) and cognitive performance on each of the novel BHA tests controlling for CDR Sum of Boxes, clinical phenotype (dummy-coded: 0 = non-amnestic MCI/atypical dementia, 1 = amnestic MCI/amnestic-predominant dementia), and time difference between PET acquisition and BHA completion. Tau analyses were not performed with the MoCA due to a small sample size (*n* = 39) of participants who completed this measure.

Logistic and linear regression models were performed in R (v4.0.2, R Project for Statistical Computing) with two-tailed significance level for regression models set at *P* < .05. All models were checked for overdispersion, influential values, and multicollinearity. We report *P* values without adjusting for multiple comparisons as this methodology focuses on avoiding one or more results with *P* < .05 in the case where all differences are truly zero, which is likely an unrealistic hypothesis in our situation. In addition, adjustment would require that each result detract from the others, but there are known biological relationships among the measures considered here, and these allow consistent findings to support each other instead of detracting from one another. Thus, we use scientific judgment rather than formal methods of adjustment to indicate where caution is warranted despite findings with *P* < .05.

## Results

### Sample characteristics

Demographic characteristics of Aβ PET sample are presented in Table 1. Aβ+ sample was comprised of 64 participants with MCI (36 amnestic, 28 non-amnestic [see Supplementary Table 2 for detailed description of clinical phenotypes]) and 33 participants with dementia (21 amnestic-predominant AD-type dementia, 2 frontal variant AD-type dementia, 1 behavioral variant frontotemporal dementia [bvFTD], 2 corticobasal syndrome [CBS], 2 logopenic variant primary progressive aphasia [PPA], 3 posterior cortical atrophy [PCA], 1 unspecified PPA, 1 progressive supranuclear palsy [PSP]). Aβ− sample consisted of 29 participants with MCI (12 amnestic, 17 non-amnestic [Supplementary Table 2]) and 14 participants with dementia (2 amnestic-predominant AD-type dementia, 2 frontal variant AD-type dementia, 6 bvFTD, 1 CBS, 1 dementia with Lewy bodies [DLB], 1 non-fluent variant PPA, 1 PSP). Aβ+ participants were more likely to have an amnestic clinical phenotype, had poorer performance on Favorites and Match tests, and greater BHA-ECog scores compared to the Aβ− group (Table 1). Table 2 presents baseline characteristics of the tau-PET subsample, which was comprised of 32 MCI (13 amnestic, 19 non-amnestic [Supplementary Table 2]) and 28 dementia (20 amnestic-predominant AD-type dementia, 2 frontal variant AD-type dementia, 2 logopenic variant PPA, 3 PCA, 1 unspecified PPA) participants. Demographic characteristics of Aβ+ participants who did not complete tau-PET are also reported in Table 2.

**Table 1:** Demographic characteristics of the Aβ PET sample (*N* = 140)

	Aβ− (***n*** = 43)	Aβ+ (***n*** = 97)	***t / odds ratio*** ***[95% CI]***	***P***
Age	66.6 (11.6)	68.6 (8.7)	0.98 [− 2.01; 5.91]	.33
Education	16.4 (2.9)	16.9 (2.6)	0.97 [− 0.52; 1.53]	.33
Female	17 (40%)	43 (44%)	1.22 [0.55; 2.72]	.71
Non-Hispanic White	38 (88%)	85 (88%)	0.93 [0.24; 3.10]	.99
MCI	29 (67%)	64 (66%)	1.07 [0.47; 2.50]	.99
Amnestic phenotype	14 (33%)	57 (59%)	2.93 [1.31; 6.81]	.006
CDR-SB	2.3 (2.1)	3.1 (2.2)	1.98 [− 0.01; 1.52]	.05
Time difference (yrs)	0.3 (0.3)	0.4 (0.6)	1.74 [− 0.02; 0.30]	.08
Favorites *z*-score	− 1.3 (1.3) *n = 38*	− 2.4 (1.1) *n = 88*	− 4.42 [− 1.54; − 0.58]	< .001
Match *z*-score	− 2.0 (1.4) *n = 42*	− 3.6 (2.7) *n = 93*	− 4.81 [− 2.38; − 0.99]	< .001
Line Orientation *z*-score	− 0.6 (1.2) *n = 41*	− 1.2 (2.6) *n = 94*	− 1.79 [− 1.20; 0.06]	.08
BHS-ECog score	2.0 (0.7) *n = 38*	2.5 (0.7) *n = 85*	2.94 [0.14; 0.71]	.005

Data are presented as mean (standard deviation) for continuous variables and *n* (% of the total sample) for categorical variables. Time difference represents the years between the BHA completion and PET acquisition presented in absolute values. For Brain Health Assessment measures, the number of participants with complete data are included. *P* values are based on independent sample *t*-tests for continuous variables and Fisher’s exact tests for categorical variables between Aβ− and Aβ+ groups. The 95% confidence intervals (CI) are reported for mean differences for *t*-tests and odd ratios for Fisher’s exact tests. *BHS-ECog* Brain Health Survey Everyday Cognition Scales, *CDR-SB* Clinical Dementia Rating Scale Sum of Boxes, *MCI* mild cognitive impairment, *PET* positron emission tomography, *yrs*. years

**Table 2:** Demographic characteristics of the Aβ+ participants with and without tau-PET

	Tau-PET (***n*** = 60)	No tau-PET (***n*** = 37)	***t / odds ratio*** ***[95% CI]***	***P***
Age	67.1 (9.2)	70.4 (6.8)	− 2.06 [− 6.61; − 0.12]	.04
Education	16.4 (2.4)	17.6 (2.8)	− 2.01 [− 2.23; − 0.01]	.05
Female	32 (53%)	13 (35%)	1.96 [0.79; 5.04]	.14
Non-Hispanic White	56 (93%)	32 (86%)	1.18 [0.27; 4.75]	.76
MCI	32 (53%)	31 (84%)	4.17 [1.43; 14.06]	.004
Amnestic phenotype	33 (55%)	24 (65%)	0.66 [0.26; 1.66]	.40
CDR-SB	4.3 (2.2)	2.3 (1.7)	2.98 [0.40; 2.01]	.004
Time difference (yrs)	0.2 (0.3)	0.7 (0.9)	− 2.67 [− 0.70; − 0.10]	.01
Favorites *z*-score	− 2.7 (1.0) *n = 51*	− 1.9 (1.1) *n = 36*	− 3.17 [− 1.22; − 0.28]	.002
Match *z*-score	− 4.6 (2.5) *n = 54*	− 2.3 (2.3) *n = 37*	− 4.28 [− 3.19; − 1.17]	< .001
Line Orientation *z*-score	− 1.5 (2.6) *n = 56*	− 0.5 (2.0) *n = 37*	2.15 [− 2.04; − 0.08]	.03
BHS-ECog score	2.6 (0.6) *n = 50*	2.3 (0.7) *n = 33*	1.58 [− 0.07; 0.58]	.12

Data are presented as mean (standard deviation) for continuous variables and *n* (% of the total sample) for categorical variables. Time difference represents the years between the BHA completion and PET acquisition presented in absolute values. For Brain Health Assessment measures, the number of participants with complete data is included. *P* values are based on independent sample *t*-tests for continuous variables and Fisher’s exact tests for categorical variables between groups with and without tau-PET. The 95% confidence intervals (CI) are reported for mean differences for t-tests and odd ratios for Fisher’s exact tests. *BHS-ECog* Brain Health Survey Everyday Cognition Scales, *CDR-SB* Clinical Dementia Rating Scale Sum of Boxes, *MCI* mild cognitive impairment, *PET* positron emission tomography, *yrs.* years

### Associations of BHA tests with Aβ PET

**Fig. 2:**
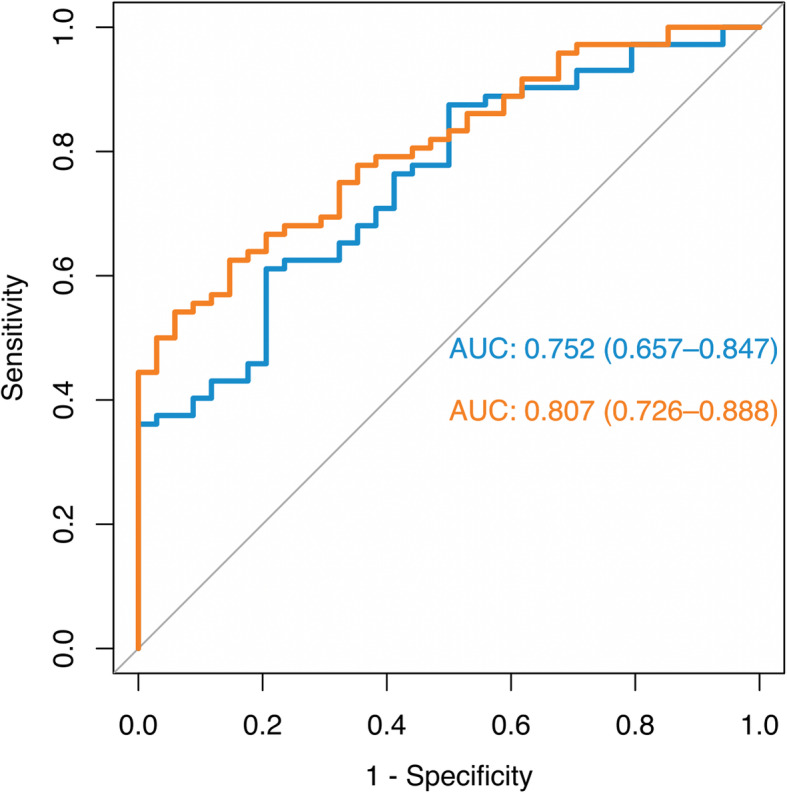
Receiver operating characteristic curves predicting Aβ+ PET status. Legend: Blue lines are based on BHA measures only (Favorites, Match, and BHS-ECog) and orange lines are based on the BHA measures and an amnestic clinical phenotype. Abbreviations: BHA, Brain Health Assessment

Logistic regression results showed significant associations between Favorites, Match, and BHS-ECog scores and Aβ+ status (Table 3). Among other predictors, only an amnestic clinical phenotype was associated with Aβ positivity (Table 3). Sensitivity analyses using unadjusted BHA test scores showed similar results (Supplementary Table 3). Figure 2 illustrates ROC curves showing accuracy in predicting Aβ+ status based on BHA measures alone (Favorites, Match, and BHS-ECog) and with addition of an amnestic clinical phenotype. Results of the likelihood ratio test comparing goodness of fit between unadjusted and adjusted models revealed a significantly better fit of the adjusted model (*χ*^2^ = 9.47, *P* = .002). The MoCA also discriminated between Aβ− and Aβ+ groups but with lower accuracy (Supplementary Table 3, Supplementary Fig. 1).

**Table 3:** Results of logistic regression analyses predicting Aβ+ PET status

	B	SE	***z***	***P***
**Favorites**				
Age	0.022	0.026	0.83	.41
Female	0.397	0.474	0.84	.40
Education	0.074	0.084	0.88	.38
CDR-SB	0.081	0.122	0.67	.51
Amnestic phenotype	0.890	0.479	1.86	.06
Time difference (yrs)	0.688	0.474	1.45	.15
Favorites *z*-score	− 0.673	0.196	− 3.44	< .001
**Match**				
Age	0.035	0.024	1.47	.14
Female	− 0.034	0.459	− 0.07	.94
Education	0.138	0.090	1.54	.12
CDR-SB	0.022	0.128	0.17	.86
Amnestic phenotype	1.465	0.460	3.18	.001
Time difference (yrs)	0.488	0.418	1.17	.24
Match *z*-score	− 0.521	0.136	− 3.82	< .001
**Line Orientation**				
Age	0.024	0.023	1.04	.30
Female	0.266	0.428	0.62	.53
Education	0.099	0.084	1.18	.24
CDR-SB	0.184	0.112	1.64	.10
Amnestic phenotype	1.475	0.442	3.34	< .001
Time difference (yrs)	0.571	0.439	1.30	.19
Line Orientation *z*-score	− 0.257	0.136	− 1.89	.06
**BHS-ECog**				
Age	− 0.001	0.027	− 0.01	.99
Female	0.496	0.465	1.07	.29
Education	0.074	0.083	0.89	.38
CDR-SB	0.076	0.134	0.57	.57
Amnestic phenotype	1.295	0.486	2.66	.008
Time difference (yrs)	0.419	0.426	0.98	.33
BHS-ECog Score	0.864	0.398	2.17	.03

Abbreviations: *B* log odds, *BHS-ECog* Brain Health Survey Everyday Cognition Scales, *CDR-SB* Clinical Dementia Rating Scale Sum of Boxes, *MCI* mild cognitive impairment, *PET* positron emission tomography, *SE* standard error, *yrs*. years

### Associations of BHA tests with tau PET

**Fig. 3:**
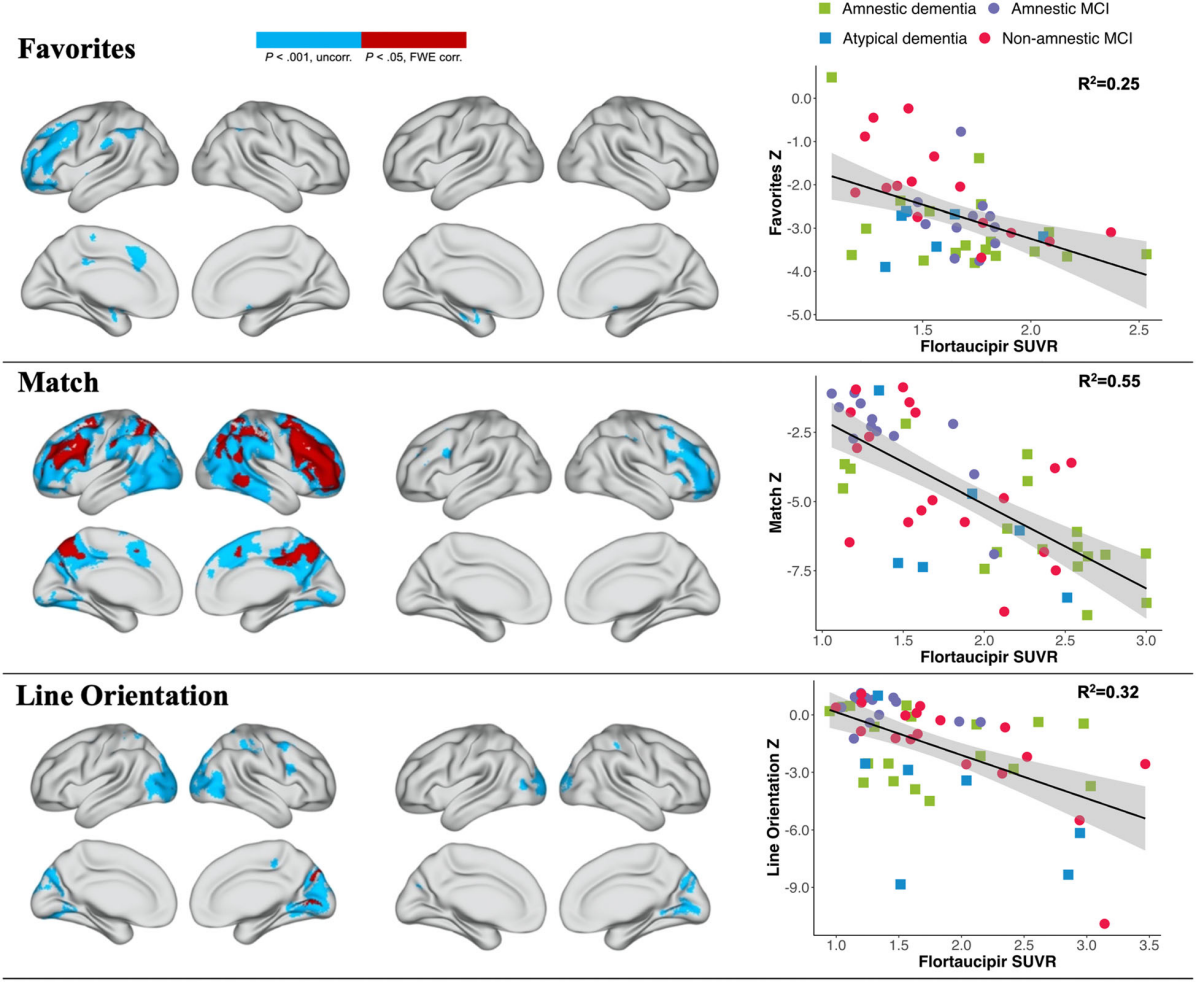
Voxel-wise results of independent regressions between BHA tests and Flortaucipir PET SUVR. Legend: Left column shows voxel-wise associations between individual BHA tests and Flortaucipir SUVR without covariates. Middle column shows voxel-wise associations between individual BHA tests and Flortaucipir SUVR with inclusion of age as a covariate. Voxel-wise associations are shown at uncorrected *P* < .001 in blue and at FWE-corrected *P* < .05 in red. Right column illustrates scatter plots and modeled regression lines (including covariates of CDR Sum of Boxes, clinical phenotype [dummy-coded: 0 = non-amnestic MCI/atypical dementia, 1 = amnestic MCI/amnestic-predominant dementia], and time difference between PET acquisition and BHA completion) on the associations between individual BHA tests and Flortaucipir SUVR in significant clusters at *P* < .001 uncorrected threshold. Abbreviations: BHA, Brain Health Assessment; CDR-SB, Clinical Dementia Rating Scale Sum of Boxes; FWE, family-wise error; MCI, mild cognitive impairment; PET, positron emission tomography; SUVR, standard uptake value ratio

Results of voxel-wise regression analyses of individual BHA tests and tau SUVR are presented in Fig. 3. After controlling for age, Favorites scores were associated with Flortaucipir SUVR in mesial temporal lobes, Match scores with Flortaucipir SUVR in frontal and parietal lobes, and Line Orientation performance with Flortaucipir SUVR in occipital and parietal lobes (Fig. 3). Sensitivity analyses including additional covariates for sex and education showed similar results (Supplementary Fig. 2). The association with Flortaucipir SUVR in significant clusters (at *P* < .001 uncorrected threshold) was strongest for Match (*R*^2^ = .55), followed by Line Orientation (*R*^2^ = .32) and Favorites (*R*^2^ = .25; Fig. 3).

## Discussion

Our findings suggest that individual BHA cognitive measures are significantly associated with both in vivo AD pathological markers and support use of highly sensitive and reliable brief cognitive measures to help identify and monitor patients with suspected AD pathology in clinical practice. A particular strength of our findings is the brevity of the BHA battery, which takes only 10 min to administer [29, 30] making it highly feasible for widescale implementation in busy clinical settings. Also, given its computerized nature, the BHA does not require administration by a trained specialist or manual scoring of results and features an automated comprehensive reporting system to facilitate interpretation of results by non-specialists.

Specifically, we found significant associations between individual BHA tests of associative memory and processing speed and executive functions and Aβ positivity in both MCI and dementia. These findings are largely consistent with prior reports on the associations of memory and executive measures with Aβ burden [19–21] in clinically mixed samples, and with our prior results on these tests being associated with regional gray matter volumes typically affected in the early symptomatic stages of AD [29]. While not directly comparable due to differences in cognitive measures used in the analyses, the accuracy of classification in our study is similar to or better than previously published findings on Alzheimer’s Disease Assessment Scale-Cognitive Subscale (ADAS-Cog) [19] and ADNI cognitive battery [20, 21]. Additionally, we found that the BHA measures were associated with Aβ positivity after controlling for age, sex, education, time gap, disease severity, and amnestic phenotype, which supports the notion that these measures are sensitive to Aβ deposition beyond the effects of demographic and clinical characteristics.

We also found significant associations between performance on all three novel BHA cognitive measures and regional tau SUVR among Aβ+ participants. These results are particularly important given the need for novel cognitive measures which, beyond clinical validity, also exhibit associations with brain regions affected in neurodegenerative diseases and are capable of capturing cognitive impairment associated with greater biomarker burden [27]. To our knowledge, only one other computerized cognitive measure, the National Institutes of Health Toolbox Cognition Battery (NIHTB-CB) has been previously shown to be associated with tau PET, also supporting the association between tasks of executive functions and processing speed with tau burden in cognitively unimpaired older adults [38]. Additionally, our findings are consistent with prior reports on the associations between regional tau retention and specific cognitive domains [12] and further highlight the feasibility of using brief and robust cognitive measures as indicators of potential AD-related pathological changes addressing the shortcomings of cost- and time-prohibitive neuropsychological batteries.

Our findings are also important to consider in the context of rapid developments in blood-based biomarkers for AD [39], which are also aimed to address the barriers to clinical implementation of molecular neuroimaging, including its high cost and invasiveness. Among those, plasma amyloid (Aβ_1–42_) [40] and phosphorylated tau (p-tau-181 [41, 42] and p-tau-217 [42]) have shown significant associations with corresponding PET findings. Thus, a combination of a brief cognitive assessment and a plasma test represent a promising alternative to PET imaging procedures in clinical practice and may not only help streamline case identification but also increase accessibility to appropriate interventions and clinical trials. Additionally, multimodal frontline identification would support more efficient distribution of healthcare resources and help avoid unnecessary costs to public healthcare systems.

### Limitations

Our study had a number of limitations. First, our sample was relatively small and was primarily comprised of highly educated, English-speaking non-Hispanic White individuals, which may limit generalizability of the results to other populations. Second, our sample included a substantial number of participants with less typical dementia syndromes, including earlier onset and atypical variants of AD. At the same time, this limitation may also be regarded as a strength of the study given a shift towards biological definition of AD and the importance and challenges of identifying its atypical variants in clinical practice [3, 43]. Finally, there was a trend for greater CDR Sum of Boxes scores in Aβ+ versus Aβ− participants, and although all analyses controlled for this variable, it is possible that our findings may in part be related to an overall greater a disease severity effect not captured by the CDR. Thus, current findings require replication in larger, more diverse cohorts as well as cross-validation in out-of-sample populations. Future studies should also examine longitudinal associations between BHA tests and AD PET biomarkers. Lastly, our findings should be interpreted with a caveat that applies to all studies of brain-behavior relationships: the brain bases of cognitive performance are multifactorial, and different individuals may fail the same test for different reasons.

## Conclusions

Our results showed that performance on the BHA measures is significantly associated with in vivo Aβ and regional tau PET burden in a clinically heterogeneous sample of individuals with MCI and dementia. These findings demonstrate potential for clinical applicability of brief and sensitive cognitive measures for the frontline identification of patients with underlying AD pathology. Potential implementation of the BHA or similar tools in clinical settings may support progress towards precision medicine and targeted interventions in AD research and therapies.

## Supplementary Information

**Additional file 1.**

## Data Availability

The datasets generated and/or analyzed during the current study are not publicly available due to considerations related to protection of participants’ confidentiality but are available from the corresponding author on reasonable request.

## Abbreviations

Aβ: β-amyloid
AD: Alzheimer’s disease
AUC: Area under the curve
BHA: Brain Health Assessment
BHS: Brain Health Survey
BHS-ECog: Brain Health Survey Everyday Cognition Scales
CDR: Clinical Dementia Rating
MCI: Mild cognitive impairment
ROC: Receiver operating characteristic
SUVR: Standard uptake value ratio
