# Supplementary material for: Detecting Alzheimer’s disease biomarkers with a brief tablet-based cognitive battery: sensitivity to Aβ and tau PET
Source: Alzheimers Res Ther. 2021 Feb 8;13:36. doi: 10.1186/s13195-021-00776-w (PMC7871372; doi:10.1186/s13195-021-00776-w)
Supplement: Supplementary file 1 — Additional file 1. [file 13195_2021_776_MOESM1_ESM.docx]

**Detecting Alzheimer’s disease biomarkers with a brief tablet-based cognitive battery: sensitivity to Aβ and tau PET**

**Additional files**

Elena Tsoy, Amelia Strom, Leonardo Iaccarino, Sabrina J. Erlhoff, Collette A. Goode, Anne-Marie Rodriguez, Gil D. Rabinovici, Bruce L. Miller, Joel H. Kramer, Katherine P. Rankin, Renaud La Joie, Katherine L. Possin.

**Supplementary Table 1.** Baseline demographic characteristics of the Aβ-PET subsample who completed the MoCA (N = 109).

|  | **Aβ-**  **(n = 37)** | **Aβ+**  **(n = 72)** | ***t / odds ratio***  ***[95% CI]*** | ***P*** |
| --- | --- | --- | --- | --- |
| Age | 65.9 (12.1) | 69.8 (8.3) | 1.80 [-0.45; 8.42] | .08 |
| Education | 16.3 (2.9) | 17.2 (2.6) | 1.55 [-0.25; 2.02] | .13 |
| Female | 14 (38%) | 28 (39%) | 1.05 [0.43; 2.59] | .99 |
| Non-Hispanic White | 32 (86%) | 62 (86%) | 0.97 [0.24; 3.44] | .99 |
| MCI | 23 (62%) | 52 (72%) | 0.63 [0.25; 1.61] | .38 |
| Amnestic phenotype | 13 (35%) | 42 (58%) | 2.56 [1.06; 6.44] | .03 |
| CDR-SB | 2.5 (2.1) | 2.7 (1.9) | 0.60 [-0.58; 1.08] | .55 |
| Time difference (yrs) | 0.3 (0.3) | 0.5 (0.6) | 2.00 [0.00; 0.36] | .05 |
| MoCA | 22.2 (3.6) | 20.3 (4.9) | -2.42 [-3.60; -0.36] | .02 |

Data are presented as mean (standard deviation) for continuous variables and n (% of the total sample) for categorical variables. Time difference represents the years between the MoCA completion and PET acquisition presented in absolute values. *P* values are based on independent sample t-tests for continuous variables and Fisher’s exact tests for categorical variables between Aβ- and Aβ+ groups. 95% confidence intervals (CI) are reported for mean differences for t-tests and odd ratios for Fisher’s exact tests. CDR-SB, Clinical Dementia Rating Scale Sum of Boxes; MCI, mild cognitive impairment; PET, positron emission tomography; yrs, years.

**Supplementary Table 2.** Description of MCI non-amnestic phenotypes by Aβ status.

| **Aβ- (n = 17)** | **Aβ+ (n = 28)** | **Aβ+ tau sample (n = 19)** |
| --- | --- | --- |
| 7 frontal/executive type of unknown etiology  6 non-fluent variant primary progressive aphasia  2 corticobasal syndrome  1 semantic variant primary progressive aphasia  1 due to suspected traumatic encephalopathy syndrome | 9 frontal/executive type of unknown etiology  7 logopenic variant primary progressive aphasia  6 posterior cortical atrophy  3 unspecified primary progressive aphasia  2 progressive supranuclear palsy  1 semantic variant primary progressive aphasia | 7 logopenic variant primary progressive aphasia  6 posterior cortical atrophy  3 frontal/executive type of unknown etiology  2 unspecified primary progressive aphasia  1 semantic variant primary progressive aphasia |

**Supplementary Table 3.** Results of logistic regression analyses predicting Aβ+ PET status using demographically unadjusted Brain Health Assessment scores and the MoCA.

|  | **B** | **SE** | ***z*** | ***P*** |
| --- | --- | --- | --- | --- |
| **Favorites (n = 126)** |  |  |  |  |
| Age | -0.006 | 0.026 | -0.22 | .83 |
| Female | 0.617 | 0.474 | 1.30 | .19 |
| Education | 0.098 | 0.085 | 1.15 | .25 |
| CDR-SB | 0.077 | 0.123 | 0.62 | .53 |
| Amnestic phenotype | 0.882 | 0.481 | 1.83 | .07 |
| Time difference (yrs) | 0.680 | 0.437 | 1.44 | .15 |
| Favorites raw score | -0.148 | 0.042 | -3.52 | <.001 |
| **Match (n = 135)** |  |  |  |  |
| Age | -0.006 | 0.024 | -0.23 | .82 |
| Female | 0.125 | 0.451 | 0.28 | .78 |
| Education | 0.138 | 0.090 | 1.54 | .12 |
| CDR-SB | 0.022 | 0.128 | 0.17 | .86 |
| Amnestic phenotype | 1.465 | 0.460 | 3.18 | .001 |
| Time difference (yrs) | 0.488 | 0.418 | 1.17 | .24 |
| Match raw score | -0.089 | 0.023 | -3.82 | <.001 |
| **Line Orientation (n = 135)** |  |  |  |  |
| Age | 0.019 | 0.023 | 0.84 | .40 |
| Female | 0.172 | 0.437 | 0.39 | .69 |
| Education | 0.099 | 0.084 | 1.18 | .24 |
| CDR-SB | 0.184 | 0.112 | 1.64 | .10 |
| Amnestic phenotype | 1.475 | 0.442 | 3.34 | <.001 |
| Time difference (yrs) | 0.571 | 0.439 | 1.30 | .19 |
| Line Orientation raw score^*^ | 0.095 | 0.050 | 1.89 | .06 |
| **MoCA (n = 109)** |  |  |  |  |
| Age | 0.021 | 0.024 | 0.86 | .39 |
| Female | 0.080 | 0.462 | 0.17 | .86 |
| Education | 0.167 | 0.096 | 1.74 | .08 |
| CDR-SB | -0.120 | 0.124 | -0.16 | .87 |
| Amnestic phenotype | 0.904 | 0.465 | 1.94 | .05 |
| Time difference (yrs) | 0.605 | 0.412 | 1.47 | .14 |
| MoCA | -0.156 | 0.063 | -2.49 | .01 |

^*^Greater raw values indicate worse performance.

Abbreviations: B, log odds; CDR-SB, Clinical Dementia Rating Scale Sum of Boxes; MCI, mild cognitive impairment; PET, positron emission tomography; SE, standard error; yrs, years.

**Supplementary Figure 1.** Receiver operating characteristic curves predicting Aβ+ PET status using the MoCA (n = 109).


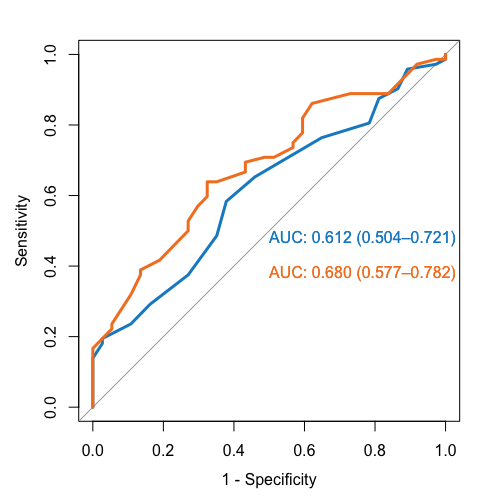


Blue lines are based on MoCA total score only and orange lines are based on the MoCA and an amnestic clinical phenotype.

**Supplementary Figure 2.** Voxel-wise results of independent regressions between BHA tests and Flortaucipir-PET SUVR with and without inclusion of demographic covariates.

SPM “glass brains” showing significant voxels (at an uncorrected *P*<.001 threshold) that showed an association between tau-PET and each of the BHA cognitive measures (3 rows) without any covariates (left column), controlling for age (middle), and controlling for age, sex, and education (right).
